# “Under the Skin” and into the Gut: Social Epidemiology of the Microbiome

**DOI:** 10.1007/s40471-018-0167-7

**Published:** 2018-09-20

**Authors:** Jennifer Beam Dowd, Audrey Renson

**Affiliations:** 1 Department of Global Health and Social Medicine, King’s College London, The Strand, London, WC2R 2LS UK; 2 Epidemiology and Biostatistics, CUNY Graduate School of Public Health and Health Policy, 55 W 125th St, New York, NY 10027 USA; 3 Department of Epidemiology, Gillings School of Global Public Health, University of North Carolina at Chapel Hill, 135 Dauer Dr, Chapel Hill, NC 27599 USA

**Keywords:** Microbiome, Socioeconomic factors, Health disparities, Psychosocial stress, Social epidemiology, Population health, Built environment, Social relationships, Socioeconomic status, Race/ethnicity

## Abstract

**Purpose of the Review:**

As the science of the microbiome advances, social epidemiologists can contribute to understanding how the broader social environment shapes the microbiome over the life course. This review summarizes current research and describes potential mechanisms of the social epidemiology of the microbiome.

**Recent Findings:**

Most existing literature linking the social environment and the microbiome comes from animal models, focused on the impact of social interactions and psychosocial stress. Suggestive evidence of the importance of early life exposures, health behaviors, and the built environment also point to the importance of the social environment for the microbiome in humans.

**Summary:**

Social epidemiology as a field is well poised to contribute expertise in theory and measurement of the broader social environment to this new area, and to consider both the upstream and downstream mechanisms by which this environment gets “under the skin” and “into the gut.” As population-level microbiome data becomes increasingly available, we encourage investigation of the multi-level determinants of the microbiome and how the microbiome may link the social environment and health.

## Introduction

Social and biological processes interact across the life course to produce health outcomes, including persistent health inequalities by socioeconomic status (SES) [1]. Even while keeping their eye keenly on the upstream determinants of these health inequalities, social epidemiologists’ interest in how social conditions “get under the skin” has grown rapidly over the past two decades, leading to novel insights into the biology of disadvantage ranging from cortisol responses to epigenetic gene expression [2, 3]. As biological science advances, social epidemiology can leverage this experience to conceptualize and measure how the social environment shapes new areas of biology. This review focuses on the new science of the human microbiome—the trillions of microbes that inhabit the human body and their genes—that are believed to have profound implications for human health [4]. Indeed, we are estimated to have at least as many microbial cells as human cells in our body [5], challenging traditional notions of the human “self” and pushing us to understand how humans interact with microbes throughout our lives [6]. Early findings of racial/ethnic and socioeconomic variation in the gut, oral, and vaginal microbiome [7–13, 14•] have led to calls for investigation into the potential role of the microbiome in health disparities [15].

**Fig. 1:**
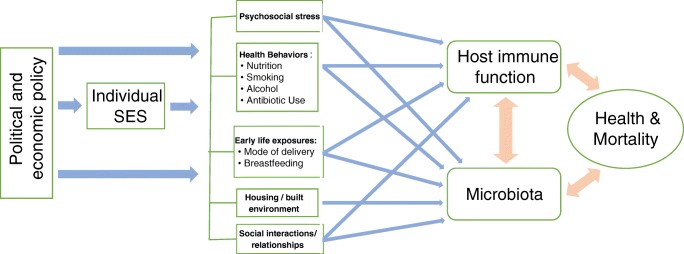
Mechanisms linking the social environment and the microbiome

We underscore the need for robust inquiry into the social epidemiology of the microbiome in the early days of this new scientific area. The mechanisms through which social and demographic factors shape the microbiome over the life course are not well understood, but their importance has been highlighted by recent findings that genetic factors explain little variation in the gut microbiome, leaving “environmental” factors as the predominant determinant [16•]. But what constitutes the “environment” with respect to the microbiome? A broader consideration of how the social, physical, and psychosocial environments shape the microbiome over the life course is needed to understand individual and population level variation in the microbiome and ultimately how to intervene on it [17]. In this review, we assess the nascent research on potential mechanisms linking social factors to the microbiome including early life exposures, psychosocial stress, social relationships, the built environment, health behaviors, and socioeconomic status (outlined in Fig. 1) and suggest the most promising areas for future investigation.

## Early Life

Birth and early post-partum life are critical periods for microbiome acquisition and development, and periods strongly influenced by one’s social environment. Whether organisms pass into the fetus from the placenta is still an open question, though the most thorough study to date is consistent with placental sterility [18]. Babies delivered vaginally acquire bacterial communities similar to their mother’s vaginal microbiota, whereas C-section infants resemble those found on the skin surface as well as the surrounding environment [19, 20]. Exclusively breasted infants have increased taxa in their gut that are used in probiotics such as *B. longum*, while formula-fed infants have elevated levels of the more negatively connotated *C. difficile* [20, 21]. The microbiome of human breastmilk itself has been shown to differ by factors such as maternal obesity and elective vs. non-elective C-sections [22]. Using a multiple body site metagenomic approach following 25 mother-baby pairs, Ferreti et al. recently documented the strong influence of maternal-child vertical transmission on the infant gut microbiome and found that this seeding from maternal sources is a continuous process, highlighting both the importance of the composition of maternal microbiome itself as well as maternal-child interactions in the development of the infant microbiome [23••]. In the USA, older mothers, those reporting stressful life events prior to conception, and those who were obese prior to pregnancy had higher risks of non-elective C-sections, though no independent association with SES or race/ethnicity factors was found adjusting for these factors [24]. Breastfeeding initiation rates and duration vary by maternal education and race/ethnicity in the USA, with non-Hispanic black mothers the least likely to initiate and maintain breastfeeding [25]. Thus, differences in mode of delivery, feeding practices, and maternal health are all potentially important mechanisms through which the social environment shapes microbial exposures from the first day of life.

## Psychosocial Stress

A significant body of research implicates the physiologic response to chronic and repeated stressful life events in health inequalities (see [26] for a systemic review). Broadly defined, stress is a disruption to homeostasis, which can be real or anticipated, physical or psychological in nature [27]. The physiologic response to stress involves the nervous, immune, and endocrine systems; systems whose development and functioning are increasingly understood to be influenced by the gut microbiota [28]. Conversely, exposure to stress can impact the structure and function of the microbiota itself [29]; therefore, a central focus of research on social factors and the microbiome will likely be unraveling the role of socially determined stress, psychological trauma, and adverse life events, in shaping microbiota structure and function.

Indirectly, psychosocial stress has long been observed to impact gastrointestinal tract functioning, dating back at least to the nineteenth century, when reduced gastric acid secretion was observed in a gastric fistula patient during periods of fear [30].

### Stress and the Microbiome in Animal Models

The concept that stress and its mediators can directly alter the gut microbiome is more recent. Specifically, neuroendocrine mediators of stress, particularly norepinephrine, directly impact microbial bacterial growth in the gut [31], including enhancing growth of bacteria such as *E.coli* [32] and the expression of different types of virulence factors that increase the severity of the disease [33]. Communication between the brain and gut microbiota is complex and bidirectional, occurring most primarily via the vagus nerve, which enervates nearly the entire digestive tract and can receive information about the state of enteric microbial communities [34].

In mice, exposure to social stressors has been repeatedly shown to cause important alterations to the gut microbiome, in ways that alter microbiota-immune system interactions, increase susceptibility to infection, and promote inflammatory mediators [35]. For example, acute exposure to a stressor has also been shown to select for anaerobic gut organisms and decrease richness [29], and a model of chronic social defeat was likewise associated with decreased gut richness and diversity [36••]. In an early-life stress model, maternally separated mice had a compositionally altered gut microbiome relative to controls [37]. These models have also explored the role of maternal stress on offspring outcomes-maternal stress during pregnancy altered proteins related to vaginal immunity and abundance of *Lactobacillus* in dams, which in turn decreased the abundance of this bacterium in the gut microbiota of their offspring [38]. Moreover, changes to the murine gut microbiota in response to stress have clear health implications: They appear to fully mediate stress-related immune cytokine production [31] and lead to downregulation of short chain fatty acid and neurotransmitter pathways [36••], with orally administered Bifidobacterium conferring resistance to some of these effects [39]. Some of these findings have been replicated in primates as well. In captive rhesus monkeys, maternal separation stress induced reductions in lactobacilli in intestinal microflora and higher rates of opportunistic enteric infection [40]. In rhesus monkeys whose mothers were exposed to startle stressors during pregnancy, lactobacilli levels in the gut microbiota were lower during the first 6 months of life, which in turn disrupted the development of natural resistance to the enteric pathogen *Shigella flexneri* [41].

### Stress and the Microbiome in Humans

Few studies have yet examined the interplay between the microbiota and stress in humans. One study evaluated the gut microbiota of 73 soldiers before and after a multiple-stressor military training environment, finding an increased intestinal permeability, greater alpha diversity, and changes in relative abundance of > 50% of 16S taxa identified in stool samples [42]. A reduction in lactobacilli shed in stool was also found for college students during final exams when levels of perceived daily and weekly stress were higher, although the confounding effects of changing diet could not be ruled out [43]. In the oral microbiome, experimentally induced acute stressors in humans have been shown to increase the saliva-mediated adherence of microbes including *H. pylori* and *Streptoccocus gordonii*, suggesting one mechanism by which stress may affect mucosal microbiota and susceptibility to infections [44]. Recent work shows that human oral microbiome samples treated with the stress hormone cortisol display selection for oral pathogens and an altered transcriptional profile consistent with periodontal disease, suggesting direct effects as well [45].

Studies of psychosocial influences on the microbiome will clearly be a growth field in the near future, likely using experimental models in animals and humans that have been successful in understanding other areas of stress biology. One important area will be to identify the developmental periods most sensitive to stress and how reversible stress-related alterations to the microbiome might be. As microbiome data becomes available in longitudinal human population studies [46], it will be possible for social epidemiologists to examine the impact of more naturalistic social and economic stressors such as early life adversity and unemployment on the microbiome and test whether the microbiome may mediate stressful life events and health outcomes across the life course.

## Social Relationships

Social relationships are well-established predictors of overall health and mortality [47], and social interactions have long played an important role in the transmission of pathogenic microbes [48, 49]. Thus, it is likely that social interactions are important in the acquisition and maintenance of commensal microbiota. Indeed, evidence is accumulating from both animals and human studies that social organization and behavior are associated with the diversity and composition of the gut microbiota, though evidence is still mixed on the exact features of sociability that are most important.

### Social Relationships and the Microbiome in Non-human Primates

In one of the first studies of its kind, Tung et al. found that social group membership among baboons in Kenya predicted the taxonomic structure and function of the gut microbiome, even taking account of diet, kinship, and shared environments [50••]. The authors suggested this as evidence of direct transmission of microbes through physical contact with social partners. Grienesen et al. extended this analysis on the same groups of animals, testing measures of alpha and beta diversity along different dimensions of social group and organization [51]. They confirmed that members of the same social group had more similar gut microbiomes and identified this for both the core (more stable) and non-core (more variable) microbiome. More diverse gut microbiomes are believed to be “healthier” than less diverse microbes, providing stability and redundancy within the system, and more social contacts may contribute to this diversity [52]. Members of the larger of the two social groups were found to have higher alpha diversity in their guts, but this was not true for individuals with the most “social grooming” partners, suggesting in contrast to the Tung et al. conclusion that indirect transmission of microbes may be more important for shaping diversity than direct transmission via physical contact.

Similar associations have been identified in other primate species. Utilizing 8 years of behavioral observations from chimpanzees in Tanzania, Moeller et al. found evidence that increased social contact contributed to higher diversity of the gut microbiome at the individual level but contributed to increased similarity among interacting chimps at the group level [53]. Again, these increases in similarity did not seem to be due to the consumption of more similar diets and are believed to result from direct contact or indirect transfer from feces deposited in the environment. Moeller et al. also found that the inheritance of gut microbial communities across generations appeared not to happen vertically from parent to offspring but rather from horizontal transfer from socially interacting hosts, with similarities among unrelated group members similar to those of family members. There has been variation in findings across studies and species—Amato et al. for instance found among black howler monkeys that closely related individuals had marginally *less* similar gut microbial communities than non-related individuals, but those who spent more time in direct contact and close proximity had more similar gut microbial communities [54].

Raulo et al., using social network analysis of red bellied lemurs, found that family group identity was the most important factor explaining variation in gut microbial profiles [55]. Associations between breeding pairs were as similar as offspring, suggesting that these similarities are not primarily due to shared genetics. Contrary to expectations, they found that group size was not correlated with alpha diversity, and individual sociability was negatively correlated with alpha diversity. They speculate that this could be due to confounding by stress, which lowers diversity and increases affiliative behavior in primates, or possibly due to enrichment of certain bacteria within a given community that lowers overall weighted alpha diversity measures. In another wild primate study of Verreaux’s sifaka (a medium-sized primate in the lemur family), Perofsky et al. show that social groups with denser grooming networks have more homogeneous gut microbial communities, and the most gregarious individuals within social groups have the greatest microbial diversity [56]. Interestingly, Grienesen et al. also found that “immigrant” male animals who had lived longer with their social group had more similar microbiomes to the other group members than more recent arrivals to the group. Given that changes in the microbiome due to shared diet are believed to take place in a matter of days [57], this was taken as evidence of other modes physical or social transmission. Previous work from chimpanzees found that long-term immigrants to new social groups harbored the most distinctive gut microbiota and maintained gut microbiome signatures from both groups [58].

Overall, the non-human primate literature supports the notion that gut microbial composition depends on social interactions much more than shared genetics and that direct physical contact is an important mechanism in addition to the potential role of shared diet and physical environments. Some of this literature draws on a life course ecology framework, suggesting that the benefits of social transmission of gut microbiota for enhancing immunity may have played a role in the evolution of sociality [55]. Moeller et al. notes that the social dynamics of the human pan-microbiome have not been investigated because of a “lack of longitudinal monitoring of human social groups,” highlighting an opportunity for social scientists and epidemiologists moving into this area.

### Social Relationships and the Human Microbiome

While studies of long-term social networks and the microbiome in humans are currently lacking, shorter term studies have begun to lay the groundwork for understanding how humans impact the microbiome of those around them. Humans have been found to emit upwards of 10^6^ biological particles per hour, with Meadow et al. demonstrating that individuals release their own personalized microbial cloud via airborne release [59]. They suggest that recently emitted airborne microbes might more readily colonize other humans compared to those found on surfaces since they are more likely to be physiologically active. Such opportunities for transmission indeed seem to translate into more similar microbiomes among cohabitating individuals. Using data from seven families, Lax et al. found that humans sharing homes had more similar microbial communities of the nose, feet, and hands compared to those not sharing a home, likely due to skin shedding, respiratory activity, and skin-surface contact [60]. Song et al. extended this work by surveying fecal, oral, and skin microbiota from 60 families, finding that household members shared more of their microbiota than with individuals not in their household, and this effect was stronger for the skin microbiota than for oral or fecal microbiota [61]. Dog ownership also significantly increased the shared skin microbiota in cohabiting adults. Ross et al. also found that the skin microbiome of cohabitating couples was much more similar than by chance [62], and the similarity of the oral microbiota among couples has been associated with the self-reported frequency of intimate kissing [63].

Given modern hygienic practices, the mechanism by which the gut microbiome is transferred is less intuitive, leading Shaffer et al. to test for the presence of fecal and oral microbes on the hands of members of the 73 families that were also used in the Song et al. study [64]. The authors found a “surprisingly high” incidence of fecal material on hands that could specifically be tracked to that of family members and oneself. Women who were parents had more oral microbes on their hands than non-parent women, though no difference was seen for men by parenting status. The study lends credence to the hands as an important vector for the transfer of fecal and oral microbes within families, consistent with the primate evidence that sharing of microbial composition is not exclusively driven by shared diets.

### The Built Environment

Humans are born, nurtured, educated, and live out their lives in buildings. From birth, microbes inside buildings seed, colonize, and transiently occupy our bodies. Whether intentional or not, the design of buildings mediates microbial exposures and shapes the human microbiome [65].

Humans are estimated to spend up to 90% of their time indoors in industrialized nations [66], and the quality of the indoor environment and neighborhoods in which we reside is strongly socially patterned. Indoors, we interact with microbes left on surfaces, in dust that we perturb, and emissions in the air from our breath, clothes, skin, and hair [59]. In the Lax et al. study discussed above, if the families moved, their microbial signature followed them to the new home, and individuals who left the home for several days saw a decline in their contribution to the home microbiome, suggesting a rapid and dynamic process of human influence on their microbial environment [60].

Beyond the impact of humans on the microbiome of the built environment, differences in geography, ventilation, building design, and even prior flood damage can impact the types of bacteria and fungi found within homes [67]. Barberan et al. investigated the fungi and bacteria found in the dust of 1200 homes in the continental USA with a broad range of home designs, degrees of urbanization, and climatic zones. Compared to fungal community composition, bacterial communities were less associated with geographical location and climatic variables, and more dependent on the occupants of the home, particularly whether a home had dogs or cats. Using a machine learning technique, the authors could predict with 92% accuracy whether a home had a dog based on the indoor bacterial phylotypes alone, highlighting this predictable influence of pets on the home. While the sociodemographics of pet ownership are not well-characterized, a UK study found that those with the highest education levels are less likely to own pets [68]. Barberan et al. also found that the total number of inhabitants and the female/male ratio of occupants was associated with microbial composition. Two skin associated taxa (*Corynebacterium* and *Dermabacter*) and one fecal-associated taxa (*Roseburia*) were relatively more abundant in homes with fewer women, possibly driven by differences in body size and hygiene practices. *Lactobacillus*, associated with lower risk of allergies and asthma, was more abundant in homes with women. Miletto et al. investigated airborne bacteria in 29 homes in the San Francisco Bay Area, finding that community composition was associated with the number of residents and pets, activity levels, frequency of cooking and vacuum cleaning, ventilation, and abundance and type of vegetation surrounding the building [69]. It is plausible that airborne microbes can enter the gut, as inhaled organisms with aero-dynamic diameter greater than 5 μm are caught in the upper respiratory tract and cleared through mucociliary clearance into the gastrointestinal system [65].

Modern environments characterized by increasing urbanization and less exposure to green space have been implicated in changes in exposure to microbes that may be altering human microbiomes over time, as well as contributing to differentials in access to green space by socioeconomic status [70]. Ruiz-Calderon et al. studied the association of architectural design and urbanization and microbial composition of homes in South America, finding that the microbial community structure differs significantly across the urbanization gradient [71]. Despite lower occupant density, “humanization” of the microbial composition of the indoor environment also increased with urbanization.

Overall, studies of built environment and the microbiome consistently find that indoor spaces often harbor unique microbial communities whose source is dominated by humans and pets. Building occupants and surfaces affect each other in both directions, and building design and operation can influence indoor microbial communities [72•]. Little is currently known about the long-term health implications of human interactions with indoor microbiota, but we expect this to be an important area for future investigation, especially as urbanization continues to increase around the world.

### Health Behaviors

Differences in health behaviors such as diet, smoking, and medication use may play an important role in mediating associations between social factors and the microbiome [73, 74]. Such behaviors are shaped by social status across the life course, are likely socially transmissible [75], and modified by social support and stress [76]. Indeed, health behaviors contribute significantly to observed socioeconomic disparities in mortality and major morbidities in developed countries [75–77], and the impact of these behaviors on the microbiome may explain some of these links.

### Nutrition

Diet is believed to be a strong determinant of gut microbiome composition and diversity, capable of altering the microbiome both rapidly [57] and in the long term [78]. In general, diets high in animal fat and protein tend to increase abundance of gut bacteria associated with systemic inflammation, reduced insulin sensitivity, and higher LDL cholesterol [79]. In contrast, fiber and resistant starch and the antioxidant polyphenols found in fresh fruits and vegetables, seeds, tea, cocoa, and wine promote beneficial commensals such as Bifidobacterium, Lactobacillus, and Eubacterium, which reduce inflammation and contribute to gut barrier formation [79]. There are well-known differences in dietary intake by socioeconomic factors and race/ethnicity in the USA [80]. Dietary fiber intake, for instance, is lower among lower income and non-Hispanic Black Americans [81], and total and saturated fat intake is higher among non-Hispanic blacks [82]. Dietary patterns also vary geography and time in ways that likely impact the microbiome at the population level, something social epidemiologists are well-positioned to explore [83].

### Tobacco

There are strong educational gradients in cigarette smoking that have grown more pronounced over time [84]. Numerous studies in mice suggest that smoking alters the gut microbiota, leading to dysbiosis, enrichment of pathogens, and an inflammatory microenvironment in the intestine (reviewed by [85]). In humans, among people with Crohn’s disease, smokers have higher Bacteroides vs. Prevotella, a pro-inflammatory feature [86], a condition which appears to reverse following cessation, along with increases in Firmicutes and Actinobacteria phyla [87]. In addition to indirect pathways including altered gastrointestinal pH gradient and oxidative stress pathways [85], tobacco may directly alter the microbiome through direct transmission [88]. Several human studies suggest that tobacco smoking also alters the oral microbiome, with structural changes consistent with increased anaerobiosis [89] along with proliferation of pathogens and decreased colonization resistance in oral biofilms [90].

### Alcohol

The relationship between alcohol and SES is complex, such that low SES individuals [91] and individuals reporting low social support [92] are less likely to drink in general, but more likely to engage in episodic heavy drinking. Similarly, alcohol use varies by racial/ethnic subgroup, with non-Hispanic Whites in the USA frequently reporting the highest rates, but individuals of non-white ethnicity experiencing more social and health harms related to drinking [93]. The impact of alcohol on the oral and gut microbiome is not as well characterized as tobacco, but evidence is beginning to emerge. A subgroup of alcoholics was found to have colonic dysbiosis characterized by lower Bacteroidetes and higher Proteobacteria (a phylum high in pathogens) and by decreased network connectivity of the microbiome, which persisted after a period of sobriety [94]. The oral microbiomes of heavy drinkers were found to have greater richness and a different microbial profile, compared to non-drinkers, in a large cross-sectional sample [95].

### Antibiotic and Other Prescription Drug Use

Social variation in medication use and how this may impact the microbiome is not yet well characterized. Studies from the USA suggest that non-Hispanic white children are *more* likely than other race/ethnicities to receive antibiotics for a viral infection [96] and that black children were less likely to receive antibiotics for an infection that justified antibiotics [97]. Opioids are also prescribed most frequently for whites and higher SES individuals [98]. Antibiotic exposure is a well-known determinant of gut microbiota characteristics, leading to depleted diversity and altered composition with lasting effect (reviewed in [99]). A study in mice showed that opioid treatment significantly altered gut microbiota composition with greater abundance of Gram-positive pathogens, lower abundance of bile-deconjugating bacteria, and lower bile acid levels, which was reversed by fecal transplantation by non-treated mice [100]. Numerous other commonly used drugs, including proton pump inhibitors (PPIs), metformin, statins, nonsteroidal anti-inflammatory drugs (NSAIDs), and antipsychotics, are all associated with changes in the gut microbiome [101]. Although the associations of intake of these medications with socioeconomic factors is not well documented, each of these drugs are largely prescribed for conditions with marked socioeconomic inequalities: diabetes, cardiovascular disease, arthritis, and mental health disorders, respectively [102, 103].

Overall, there is growing evidence that many of the health behaviors already known to be associated with social factors, especially smoking and nutrition, likely have important impacts on the microbiome. Nonetheless, there may be influences on the microbiome such as alcohol consumption and prescription drug use that operate in ways counter to traditional social gradients, with those in more advantaged groups being exposed to more negative impacts on the microbiome. It will therefore be important to understand how the interaction of multiple exposures both influence the microbiome and are shaped by social factors across the life course.

## Socioeconomic Status

The evidence presented suggests that markers of socioeconomic status, reflecting access to resources that shape exposures to the physical, social, and psychosocial environments, is likely associated with differences in the composition of the microbiome over the life course. Thus far, two studies have examined sociodemographic factors and the oral microbiome. Belstrom et al. found significant differences in the bacterial profiles of the oral microbiome by area-level socioeconomic status in the Danish Health Examination Survey (DANHES) [11]. Notably, these differences were substantial in magnitude (20% of variation), compared to no significant differences found by other salient predictors including age, gender, alcohol consumption, body mass index, or dietary intake. Renson et al. found a significant number of differentially abundant taxa by individual level education, income, and race/ethnicity in the oral microbiome of a diverse sample from the 2013–2014 New York Health and Nutrition Examination Study (NYC-HANES) [14•]. Many of the taxa identified have known associations with oral health and other chronic diseases in the direction that would be consistent with a mechanism underlying health disparities in these conditions. Only one study to our knowledge has examined associations between social factors and the gut microbiome. Miller et al. found that higher neighborhood SES was associated with greater alpha diversity in the colonic microbiota of 44 healthy volunteers from Chicago, as well as greater abundance of *Bacteroides* and a lower abundance of *Prevotella* [12]. Overall, examination of the limited population level data on socioeconomic suggests the plausibility that SES is associated with characteristics of the microbiome and that the importance of these associations for health disparities in chronic conditions should be explored.

## Conclusion

While work establishing the importance of the microbiome for human health continues apace, thus far, research on how the social environment shapes the microbiome, especially in humans, is limited. Social epidemiology as a field is well poised to contribute expertise in theory and measurement of the broader social environment to this new area, and to consider both the upstream and downstream mechanisms by which this environment gets “under the skin,” “into the gut,” and onto every other body site. Social epidemiology can also bring a much needed population perspective [104] to the study of the microbiome. Changes in population level exposures such as C-section rates, antibiotic use, food policy, and urbanization may have important influences on the microbiome across time and cohorts, something not easily elucidated through a focus on microbiology and micro-level exposures. For example, it was recently found that trehalose, a food additive whose use by commercial food industry has dramatically increased since the late 1990s, contributes to the selection of more virulent strains of the dangerous intestinal microbe *C. difficile* and may have contributed to the upsurge in *C. diff* hospital infections [105]*.* As population-level microbiome data becomes increasingly available, we encourage future investigation of the multi-level determinants of the microbiome and how the microbiome may link the social environment and health.
